# Longitudinal Patterns of Stability and Change in Tenacious Goal Pursuit and Flexible Goal Adjustment among Older People over a 9-Year Period

**DOI:** 10.1155/2017/8017541

**Published:** 2017-05-31

**Authors:** Guillaume Martinent, Nathalie Bailly, Claude Ferrand, Kamel Gana, Caroline Giraudeau, Michèle Joulain

**Affiliations:** ^1^Laboratory of Vulnerabilities and Innovation in Sport (EA 7428), Interdisciplinary Confederation of Research in Sport (FED 4272), University of Claude Bernard Lyon 1, University of Lyon, Lyon, France; ^2^Laboratory of Psychology of the Various Stages of Life (EA 2114), Université François Rabelais, Tours, France; ^3^Laboratory of Psychology, Health and Quality of Life (EA 4139), Université de Bordeaux, Bordeaux, France

## Abstract

Using the dual-process model of assimilative-tenacity (TGP) and accommodative-flexibility (FGA), the study aims to identify trajectories of TGP and FGA over five time points within a 9-year period, explore the relationships between the trajectories of TGP and FGA, and explore if participants from distinct TGP and FGA trajectories differed in indicators of well-being and depression. Latent class growth analysis was used in a five-wave longitudinal design among an older population of 747 participants over 65 years. Results highlight (1) emergence of four trajectories for flexibility (low and increasing, moderate and increasing, moderately high and stable, and high and stable trajectories) and three trajectories for tenacity (low and stable, moderate and stable, and high and decreasing trajectories), (2) that older people belonging to particular trajectories of FGA are not more likely to belong to particular trajectories of TGP, and (3) that participants from the high and decreasing TGP and high or moderately high and stable FGA trajectories were characterized by high score of perceived health, satisfaction with life, and self-esteem and low score of depression moods. These results highlight that the heterogeneity in longitudinal TGP and FGA scores throughout the life span needs to be accounted for in future research.

## 1. Introduction

The question of how older adults regulate their own development is a recurring theme in the aging, clinical, and health literature. Major theories of developmental regulation emphasize the importance of maintaining a sense of control over personal development for better psychological well-being (e.g., the motivational theory of life-span development [1]; the model of selection, optimization, and compensation [2, 3]; the dual-process model of assimilative and accommodative coping [4]). In the present study, we followed the dual-process framework proposed by Brandtstädter and colleagues [4–6]. This model distinguishes two general coping strategies: assimilative and accommodative coping. The assimilative strategies, also called tenacious goal pursuit (TGP), involve striving for goals with commitment and determination and engaging in assimilative processes to modify the environment in order to achieve one's goals. Accommodative strategies, also called flexible goal adjustment (FGA), involve adjusting goals to constraints, modifying goals when there are obstacles, or disengaging from goals when necessary. According to Brandtstädter, even if both FGA and TGP are necessary for an adaptive self-regulation, there is an age-related shift toward flexibility over tenacity. When goal pursuit exceeds resources, or aversive circumstances seem irreversible, switching from FGA to TGP contributes to maintaining a positive balance of gains and losses in later life. This helps to overcome feelings of helplessness and to regain an overall sense of efficacy, even with functional declines and losses [4–6].

However, existing studies on age differences in developmental regulation are limited by their cross-sectional design [7], and knowledge of how coping changes over the adult life span is relatively limited. Indeed, the developmental change of tenacity and flexibility in aging has demonstrated contrasting results, especially on the flexibility mode. Although most studies have shown lower levels of tenacity with age [4, 8–13], empirical evidence has been inconclusive about age differences in flexibility. Studies have found higher [4, 10, 13, 14] or stable [9, 12, 15] levels of flexibility with greater age. These mixed results could be explained by the great variability in aging processes. Individuals experiencing irreversible and uncontrollable losses such as bereavement, death of close friends, or role losses would lead to the emergence of different trajectories of FGA and TGP. However, previous studies have mainly examined FGA and TGP using analytical strategies that are limited to understanding the extent of individual differences in changes [16, 17]. As a result, the present study explored whether several kinds of older person subgroups emerged regarding the FGA and TGP trajectories over a nine-year period.

Another important point is the controversial relationship between FGA and TGP. Some authors have suggested that high scores on both modes are maladaptive, possibly because people who tend to use both strategies are faced with the dilemma of choosing between holding on or letting go [18]. Other authors have suggested that people with high scores on FGA and TGP adapt more efficiently to changing circumstances [12, 19]. Hence, it seems that FGA may be the most beneficial to well-being when combined with a degree of TGP [16, 20]. Life events could involve a diversity of adaptive processes that may call on flexibility and tenacity according to the importance of individuals' goals. Subsequently, we expected that TGP and FGA would not be necessary in opposition but could develop in tandem over time.

Given the importance of self-regulation in adaptation processes as people age, it could be particularly useful to examine whether the distinct TGP and FGA trajectories were differentially associated with indicators of well-being. Particularly, it could enable higher risk profiles to be identified for older people in need of targeted and adaptive intervention approaches. A body of work shows that goal adjustment predicts high levels of well-being in general and in the face of age-related developmental losses [5, 6]. More precisely, FGA correlates positively with health perception, positive affect, life satisfaction, sense of control, positive self-evaluation, and low depression in old age [8, 12, 21–25]. In addition, FGA has been found to dampen the effects of age on depression [7, 8]. Such effects have also been demonstrated in relation to bodily handicaps, impaired health, loss of sensory functions, and chronic pain [9, 12, 26]. Noticeably, TGP does not show such buffering effects, although—in terms of bivariate correlations—it is also positively linked to well-being in all age groups. Our study aimed to explore whether participants from distinct TGP and FGA trajectories differed, at the end of the nine-year period, in indicators of well-being.

In sum, the present study aimed to (1) identify subgroups of older people with distinct TGP and FGA trajectories over nine years, (2) explore the relationships between TGP and FGA trajectories, and (3) explore whether participants from distinct TGP and FGA trajectories differed, at the end of the nine-year period, in life satisfaction, depression moods, self-esteem, and health perception. Given that few studies examined the extent of individual differences in TGP and FGA changes, no specific hypotheses were advanced regarding the number of trajectories, their specific characteristics, and the relationships between TGP and FGA trajectories. Concerning the relationships between trajectories and outcomes indicators, in line with Brandtstädter's model and empirical research [6, 8, 9, 12, 25], we hypothesized that (a) the developmental trajectories characterized by the highest levels of FGA or the lowest scores of TGP would be related to high levels of satisfaction with life, perceived health, and self-esteem and low levels of depression and (b) the developmental trajectories characterized by the lowest scores of FGA or the highest scores of TGP would be linked to the poorest psychological adjustment.

## 2. Method

### 2.1. Participants and Procedure

This research used data from an ongoing longitudinal study on adjustment to retirement initiated in 2001 from the University of Tours (France). All participants were volunteers living in different places in France and were contacted via associations for the elderly and advertisements in specialized magazines (Malakoff Capimmec Group). Written informed consent was obtained from each individual and the completed questionnaires were returned by mail in a prepaid envelope. Anonymity was ensured by attributing an identification number to each participant. Data collection was performed every two or three years. In the first assessment in 2001, the sample of this cohort comprised 906 participants with a mean age of 72.5 years (SD = 5.89, range = 62–95). In 2001, FGA and TGP were not assessed. Thus, the present study concerns the following years. In particular, the data used in this article were collected at five time points: 2003 (T1), 2005 (T2), 2007 (T3), 2009 (T4), and 2012 (T5). In 2003, 747 participants were included in the analysis: the mean age of participants was 72.73 (SD = 5.94, range = 65–97) and there were 430 women (57.56%) and 317 men (42.44%). With regard to marital status, 56.62% of the participants (*N* = 423) were married or had a partner and 43.37% lived alone (*N* = 324); 70% of the latter were widowed (*N* = 228). The participants had an average of 9.94 years of education (SD = 2.68). Our participants had few diseases (*M* = 2.3, SD = 1.5). The number of diseases was evaluated with the Multidimensional Functional Assessment Questionnaire (Pfeiffer, 1975). This scale uses a list of 26 common diseases in older persons (such as diabetes, chronic bronchitis, hypertension, arthritis, gastrointestinal diseases, cardiovascular diseases, and cancer). The number of illnesses was measured as the total number of diseases reported by each participant. The most frequent diseases encountered were arthritis (56%), hypertension (27.7%), gastrointestinal diseases (21.5%), and cardiovascular diseases (18.5%). Our sample was similar to French national averages (INSEE, 2005) in terms of sex and marital status, but respondents had generally completed more years of education than expected for people in this age bracket. All the participants lived in their own homes.

Refusal, low cognitive performance, and death are the common reasons for attrition in prospective studies of older people. However, to investigate the potential impact of attrition, differences in variables used in this study were tested between participants who completed the Time 1 (2003) measures and participants who dropped out of the study before Time 5 (2012). Participants who dropped out were older (*p* < .001) and less educated (*p* = .019) than the others. Concerning FGA and TGP, no difference appeared in the attrition population.

### 2.2. Measures

*Flexible goal adjustment (FGA)* and* tenacious goal pursuit* (TGP) were assessed using a French version of Brandtstädter and Renner's [5] FGA and TGP scales [15]. Each dimension contains 10 items that are rated on a 5-point Likert scale. Cronbach alphas on the five time points were .70, .68, .78, .74, and .74 for FGA and .80, .75, .76, .72, and .77 for TGP, respectively (Table 1).

*Health evaluation *was measured by a single-item self-rating of overall health ranging from 1 (very poor) to 5 (excellent) [27]: “In general, would you say your health is very poor, poor, good, very good or excellent?”* Life satisfaction* (LS) was measured using the Satisfaction with Life Scale [28], which consists of 5 items rated on a 7-point Likert scale. In the present sample, Cronbach alpha was .85 for Time 5 (T5).* Self-esteem was* assessed using Rosenberg Self-Esteem Scale [29], which consists of ten items rated on a 4-point Likert scale. In the present sample, Cronbach alpha was .82 for T5. Finally,* depression moods *were measured using the Geriatric Depression Scale-15 [30]. This is the first depression screening measure to have been developed for and validated among older people [31, 32]. Cronbach alpha was .76 for T5.

### 2.3. Data Analysis

A series of latent class growth analyses (LCGA) was performed to examine the emerging trajectories of TGP and FGA [3]. Measurement invariance testing is a prerequisite to LCGA model analysis because the measurement invariance assumption ensures a comparable definition of the latent construct over time. Therefore, we tested an unconstrained model with all loadings freely estimated and a constrained model in which factor loadings were constrained to be equal across time. For FGA and TGP, unconstrained models provided good fit to the data (FGA: TLI = .951; CFI = .965; RMSEA = .035, 90% CI = .031–.038; TGP: TLI = 945; CFI = .945; RMSEA = .047, 90% CI = .043–.053). The equality constraints imposed on factor loadings across time did not affect the overall fit of the model (FGA: TLI = .951; CFI = .968; RMSEA = .035, 90% CI = .031–.039; TPG: TLI = 945; CFI = .944; RMSEA = .048, 90% CI = .043–.053). When tested the small differences between their RMSEAs were not significant (*p* > .96) [33]. The LCGAs were carried out using Mplus version 7.3 using the full information maximum likelihood (FIML) estimation of missing data [34]. FIML is an unbiased and more efficient method compared to listwise deletion [35].

Firstly, the analysis involved the careful selection of a model that accurately captured the number and shape of the trajectories describing the constructs of TGP and FGA. Two sets of analyses were performed (one for TGP and another for FGA). Starting with a model with one trajectory, the number of trajectories was increased until there was no further improvement in the model [36]. With LCGA models, there is not a single statistical indicator of good model fit. As a result, a combination of statistical indicators was used to decide on the best-fitting model: log likelihood value, Akaike Information Criterion (AIC), Bayesian Information Criterion (BIC), Adjusted BIC (ABIC), and Lo, Mendell, and Rubin Likelihood Ratio Test (LRT [37]). The model that yielded the smallest values on the AIC, BIC, and ABIC as well as the highest values on the log likelihood value indicated the best-fitting model [38]. Additionally, the LRT was used for model comparison (chi-square difference test). Individuals were inferred to belong to the subgroup for which their posterior membership probability was the highest. The average of posterior probabilities was calculated and values above .70 were taken to suggest that members of a trajectory had a highly similar longitudinal pattern of change or stability on the variable [39]. It is noteworthy that initial LCGA models included both the linear and quadratic functions for each trajectory. Because none of the quadratic parameters was significant for either the TGP or FGA emerging trajectories, quadratic parameters were dropped for the subsequent LCGA.

Secondly, a LCGA model involving simultaneously TGP and FGA trajectories enabled us to examine whether older people belonging to particular trajectories of FGA were also more likely to belong to particular trajectories of TGP. Because previous studies have shown that age and sex of individuals could affect coping [40], an initial LCGA model included both gender and age as covariates of the trajectories of TGP and FGA. However, none of the parameters pertaining to the effects of age on the trajectories of TGP and FGA was significant. Therefore, for reasons of parsimony, only gender was included in the final LCGA model. Thirdly, to validate the FGA and TGP trajectories that emerged from the LCGA, we performed independently for FGA and TGP a series of multivariate analyses of variance with perceived health, satisfaction with life, self-esteem, and depression (measured in wave 5, i.e., at the end of the nine-year period) entered as the dependent variables to explore the difference between trajectories. In the analyses, a significant multivariate effect (*p* < .05) was followed up with post hoc comparisons of group means. Partial eta-squared (*η*^2^) provided an index of effect size.

## 3. Results

### 3.1. Independent LCGAs: Number and Shape of FGA and TGP Trajectories

Descriptive statistics for the five waves are presented in Table 1. Exploring fit indices of LCGA models, Table 2 indicated that the 3-trajectory model was the best fit for TGP, whereas the 4-trajectory model was the best fit for FGA.

### 3.2. A Latent Class Growth Analysis of FGA and TGP

Including simultaneously FGA and TGP within a unique LCGA model allowed us to explore whether older people belonging to particular trajectories of FGA were also more likely to belong to particular trajectories of TGP.

#### 3.2.1. Trajectories of FGA

The estimates of the TGP and FGA trajectories are presented in Table 3. The first trajectory (64.66%) was moderately high and stable and represented participants who reported a moderately high level of FGA across the five waves (intercept = 37.54,* p* < .001, linear = .30,* p* = .12). The second trajectory (5.49%) was high and stable and constituted the participants who reported a high level of FGA across the five waves (intercept = 48.84,* p* < .001, linear = .40,* p* = .19). The third trajectory (22.76%) was moderate and increasing and constituted the participants who experienced a moderate level of FGA while exhibiting a significant increase across the five waves (intercept = 32.63,* p* < .001, linear = .62,* p* = .01). The fourth trajectory (7.10%) was low and increasing and constituted the participants who experienced a low level of FGA while exhibiting a significant increase across the five waves (intercept = 32.63,* p* < .001, linear = .62,* p* = .01). The average posterior membership probabilities of belonging to a trajectory were .74, .80, .73, and .83, respectively.

#### 3.2.2. Trajectories of TGP

The first trajectory (39.49%), labeled low and stable, constituted the participants who reported a low level of TGP across the five waves (intercept = 23.93,* p* < .001, linear = −.23,* p* = .48). The second trajectory (18.07%), labeled high and decreasing, represented participants who reported a high level of TGP while exhibiting a significant decrease across the five waves (intercept = 36.21,* p* < .001, linear = −.49,* p* = .04). The third trajectory (42.44%), labeled moderate and stable, constituted the participants who reported a moderate level of TGP across the five waves (intercept = 29.54,* p* < .001, linear = −.48,* p* = .12). The average posterior membership probabilities were .78, .84, and .69, respectively.

#### 3.2.3. Relationships between the FGA and TGP Trajectories

Table 4 presents the transition probabilities of the final LCGA model. They reflect the probability of exhibiting particular trajectories of TGP depending on FGA trajectories. Results indicate that participants characterized by a low and increasing trajectory of FGA had a probability of .72 of belonging to the low and stable trajectory of TGP. The other transition probabilities were less than .50. For instance, participants characterized by a high and stable trajectory of FGA had probabilities of .38 and .46 of belonging to the low and stable and high and decreasing trajectories of TGP.

### 3.3. Comparison of Outcomes across the FGA and TGP Trajectories

We investigated how older people in each trajectory differed according to perceived health, satisfaction with life, self-esteem, and depression moods measured at the end of the 9-year period. Results of multivariate analyses of variance conducted separately on FGA and TGP trajectories were significant for FGA, Wilks's Lambda = .82, *F*(12,641) = 4.25,* p* < .001, and *η*^2^ = .07, and TGP, Wilks's Lambda = .86, *F*(8,486) = 4.84,* p *< .001, and *η*^2^ = .07. The results of univariate analyses of variance indicated that perceived health, satisfaction with life, self-esteem, and depression moods significantly differed across the FGA trajectories, whereas satisfaction with life, self-esteem, and depression significantly differed across the TGP trajectories (Table 5). Overall, older people from the moderately high and stable and high and stable FGA trajectories reported higher levels of perceived health, satisfaction with life, and self-esteem and lower levels of depression moods than their counterparts from the two other FGA trajectories. Older people from the high and decreasing TGP trajectory reported higher scores of satisfaction with life and self-esteem than those from the other TGP trajectories, whereas participants from the low and stable TGP trajectory experienced higher levels of depression moods than those of the two other TGP trajectories (see Table 5 for more details).

## 4. Discussion

The aims of the present study were to (1) identify subgroups of older people with distinct TGP and FGA trajectories over nine years, (2) explore the relationships between TGP and FGA trajectories, and (3) explore whether participants from distinct TGP and FGA trajectories differed, at the end of the nine-year period, in health perception, life satisfaction, depression moods, and self-esteem. LCGA results provided evidence for four FGA trajectories and three TGP trajectories. The first two trajectories of flexibility, representing a majority of our sample (70%), were characterized by relatively high and stable scores of flexibility. The stability of accommodative process was also highlighted by previous studies conducted among older people [9, 12, 41], whereas studies conducted with a large age bracket (from adolescence to elderliness) [42] indicated more of an age-related increase in flexibility. However, stable trajectories only comprised individuals with relatively high scores of flexibility. These results suggest that trajectories of flexibility are also largely dependent on the degree of flexibility at baseline. In particular, older people with high scores of flexibility could already have reached a “maximum flexibility” which remains stable over time. These high scores of flexibility can be explained by personal disposition or, given the significant number of individuals belonging to stable trajectories, more certainly because they have already faced the challenge of old age. By contrast, the two other trajectories representing a smaller part of our sample (only 30%) are characterized by relatively low scores of flexibility which increase over time. These results are in line with Brandtstädter's theory which postulates that with age flexibility becomes increasingly important [4, 10, 13, 14, 42]. For these two increasing trajectories, we can assume that older people with lower scores of flexibility are becoming increasingly flexible because irreversible or unavoidable events would incite them to be more flexible.

Concerning tenacity, a large majority of our participants (almost 80%) belonged to stable trajectories with low or moderate scores of tenacity (resp., 39.5% and 42.5%). The third trajectory is characterized by participants who reported a high level of tenacity while exhibiting a significant linear decrease during the nine-year period (representing 18% of our sample). The three trajectories are congruent with the majority of studies which postulate that tenacity is less activated over time [4, 8–13, 42].

Transition probabilities between the emerging trajectories of tenacity and flexibility help to shed new light on the links between these two modes of coping. First, participants characterized by a low and increasing trajectory of FGA were also likely to belong to the low and stable trajectory of TGP (transition probability of .72). Indeed, the less flexible people are also those that are the least tenacious (72%). By contrast there is very little chance of being inflexible and very tenacious (8%). As expected, the results of LCGA indicate that both strategies could complement each other in the everyday life of older people. In other words, negative life events involve a diversity of adaptive processes that may call on different degrees of flexibility or tenacity. This confirms the hypothesis that coping in old age responds to the challenges of life circumstances and aims at keeping a balance of gains and losses in aging. Nevertheless, it seems important to note that flexibility scores were higher than tenacity scores. This means that flexibility, relative to tenacity, becomes more important over the years. Heckhausen [43] reported comparable results concerning net flexibility (difference between flexibility and tenacity).

The present study also aimed to explore whether participants from distinct TGP and FGA trajectories differed, at the end of the nine-year period, in health perception, life satisfaction, depression moods, and self-esteem. Concerning FGA, results indicate that the moderately high and stable trajectory and the high and stable trajectory of flexibility are related to the best psychological functioning. Indeed, individuals belonging to these two FGA trajectories reported high scores of perceived health, satisfaction with life, and self-esteem and low scores of depression moods at the end of the nine-year period. By adjusting goals to personal and environmental constraints, older people channel resources to new more feasible goals which participate in better psychological well-being. Furthermore, readiness to accept one's past (which is inalterable) and to disengage without regret from counterfactual life paths is an ongoing and complex process which can initially disturb psychological functioning. This may explain why the more depressive people (with scores above the cut-off of 5) were those with low scores and increasing scores of flexibility in our study. Concerning tenacity, people belonging to the high and decreasing TGP trajectory reported high scores of life satisfaction and self-esteem and low score of depression moods than those from the other trajectories. We can assume that people with high tenacity are people who continue to strive for goals with commitment and determination certainly because they still have cognitive resources available to do this, even though the trajectories are decreasing. Hence, people have good life satisfaction and self-esteem and do not experience depressed mood. It should be noted that this profile concerns a minority of our sample. The majority of our sample who had low and stable scores of tenacity had the worst psychological functioning. Being no longer able to achieve their goals, individuals lose control of their life with a form of resignation. They do not positively reappraise their current situation to engage in actions toward new feasible goals. Persisting commitment to blocked goals may indicate difficulties in switching from assimilative to accommodative modes of coping.

Although this study provides additional information on the trajectories of flexibility and tenacity among aging people, some limitations should be mentioned. The first limitation is the degree to which these results can be generalized. Our participants lived at home and had a high socioeconomic status. We can suppose that they have not yet had to deal with major health or social problems. However, this remains a supposition and a certain number of negative life events (e.g., hospitalization, death of close family member or friends) should be considered to improve understanding of trajectories of coping profiles. We lack detailed information about the antecedents of developmental change. Secondly, it would be informative to link the different trajectories to differential survival. Such information would provide a better understanding of developmental processes. Currently, we lack information regarding death of participants preventing us from conducting such an analysis. To go further, it would also be interesting to investigate which self-regulation processes (FGA or TGP) are likely to be activated in a given situation. For example, Slangen-De Kort et al. [44] used concrete problem scenarios and showed that flexibility and tenacity depended on the type of situation.

## 5. Conclusion

The analytical approach used in this study was useful in describing the heterogeneity of patterns of longitudinal change and stability of TGP and FGA and their relationship with theoretically relevant outcomes such as life satisfaction, perceived health, self-esteem, and depression. In the current demographic aging context, most studies point to a very steep increase in the dependency ratio between 2020 and 2030, implying the need for a better understanding of how people can maintain a positive outlook on their life when resources and control decrease. In this perspective, the results of the present study highlight that the heterogeneity in longitudinal assimilative and accommodative coping needs to be accounted for in future research.

## Figures and Tables

**Table 1:** Mean, standard deviation, and Cronbach alpha for TGP and FGA across the five waves.

	Time 1			Time 2			Time 3			Time 4			Time 5		
	*M*	SD	*α*	*M*	SD	*α*	*M*	SD	*α*	*M*	SD	*α*	*M*	SD	*α*
Flexible goal adjustment	37.67	5.02	.70	37.66	4.76	.68	37.55	4.72	.78	37.45	4.47	.74	37.34	4.80	.74
Tenacious goal pursuit	30.27	5.71	.80	30.08	5.73	.75	29.47	5.81	.76	28.81	5.01	.72	28.86	5.72	.77

**Table 2:** Fit indices of LCGA models with 1–5 classes for TGP and FGA.

Number of classes	1	2	3	4	5
Number of free parameters	7	10	13	16	19

*Tenacious goal pursuit*					

Log likelihood	− 6288.95	− 5974.05	**** − 5904.05	− 5874.22	− 5858.56
AIC	12591.89	11968.10	**11834.10**	11780.43	11755.12
BIC	12624.23	12014.30	**11894.14**	11854.34	11842.88
ABIC	12602.00	11982.54	**11852.86**	11803.53	11782.55
LRT	N/A^a^	629.79^*∗*^	140.00^**¥**^	59.66	31.32

*Flexible goal adjustment*					

Log likelihood	− 5988.73	− 5753.92	− 5623.85	**** − 5552.20	− 5542.40
AIC	11991.46	11527.84	11273.70	**11136.40**	11122.80
BIC	12023.65	11573.82	11333.48	**11209.98**	11210.18
ABIC	12001.42	11542.06	11292.20	**11159.17**	11149.84
LRT	N/A^a^	469.62^*∗*^	260.13^*∗*^	143.30^**∗**^	19.60^*∗*^

*Note*. AIC = Akaike Information Criterion; BIC = Bayesian Information Criterion; ABIC = Adjusted BIC; LRT = Lo, Mendell, and Rubin Likelihood Ratio Test. ^a^LRT not available for the one-class model; ^*∗*^*p* < .05;  ^*¥*^*p* = .06. Bold entries indicate the model selected.

**Table 3:** Longitudinal trajectories of TGP and FGA across the three waves.

			Intercept			Linear	
	*N*	Estimate	SE	*p*	Estimate	SE	*p*
*Flexible goal adjustment*							
Moderately high and stable	483	37.54	.71	<.001	.30	.19	.12
High and stable	41	48.84	1.00	<.001	.40	.30	.19
Moderate and increasing	170	32.63	.89	<.001	.62	.25	.01
Low and increasing	53	26.34	1.00	<.001	.75	.33	.02

*Tenacious goal pursuit*							
Low and stable	295	23.93	2.10	<.001	−.23	.33	.48
High and decreasing	135	36.21	1.62	<.001	−.55	.27	.04
Moderate and stable	317	29.54	1.94	<.001	−.48	.31	.12

**Table 4:** Transition probabilities between the emerging trajectories of TGP and FGA.

	Tenacious goal pursuit		
Flexible goal adjustment	Low and stable	High and decreasing	Moderate and stable
Moderately high and stable	.32	.23	.45
High and stable	.38	.46	.16
Moderate and increasing	.46	.12	.42
Low and increasing	.72	.08	.21

**Table 5:** Comparison of external variables across the trajectories of FGA and TGP.

Flexible goal adjustment	(1) Moderately high and stable		(2) High and stable		(3) Moderate and increasing		(4) Low and increasing		*F*(3,245)	*p*	*η* ^2^	Tukey HSD^*∗*^
trajectories	*M*	SD	*M*	SD	*M*	SD	*M*	SD				
Perceived health	3.38	.96	3.60	.99	3.00	.79	3.00	1.26	3.76	.01	.04	1 > 3
Satisfaction with life	26.68	5.10	29.00	5.86	24.75	4.77	22.25	7.31	7.13	<.001	.08	1,2 > 3,4
Self-esteem	33.99	3.91	36.13	3.38	32.23	3.91	29.55	6.00	11.00	<.001	.12	1,2 > 3 > 4
Depression moods	2.28	2.37	1.40	1.30	2.99	2.38	5.25	3.91	10.10	<.001	.11	4 > 1,2,3

Tenacious goal pursuit	(1) Low and stable		(2) High and decreasing		(3) Moderate and stable			*F*(2,246)		*p*	*η* ^2^	Tukey HSD
trajectories	*M*	SD	*M*	SD	*M*	SD						

Perceived health	3.14	.99	3.49	.97	3.23	.92		2.39		.09	.02	
Satisfaction with life	24.60	5.60	27.98	4.29	25.91	5.58		7.05		.001	.05	2 > 1,3
Self-esteem	31.78	4.39	36.00	3.32	33.03	4.07		19.36		<.001	.14	2 > 1,3
Depression moods	3.19	2.80	1.89	2.42	2.64	2.46		4.41		.01	.03	1 > 2

*Note.* ^*∗*^All Tukey HSD are significant at *p* < .05.
